# Individual Difference Variables and the Occurrence and Effectiveness of Faking Behavior in Interviews

**DOI:** 10.3389/fpsyg.2017.00686

**Published:** 2017-05-10

**Authors:** Anne-Kathrin Buehl, Klaus G. Melchers

**Affiliations:** Institut für Psychologie und Pädagogik, Universität UlmUlm, Germany

**Keywords:** selection interview, faking, personality, attitudes, cognitive ability, social skills

## Abstract

There is widespread fear that applicants can fake during selection interviews and that this impairs the quality of selection decisions. Several theories assume that faking occurrence is influenced by personality and attitudes, which together influence applicants’ motivation to show faking behavior. However, for faking behavior to be effective, interviewees also need certain skills and abilities. To investigate the impact of several relevant individual difference variables on faking behavior and interview success, we conducted two studies. In Study 1, we surveyed 222 individuals to assess different personality variables, attitude toward faking, cognitive ability, self-reported faking behavior, and success in previous interviews, and in Study 2, we assessed cognitive ability, social skills, faking behavior, and interview performance in an interview simulation with 108 participants. Taken together, personality, as well as attitude toward faking, influenced who showed faking behavior in an interview, but there was no evidence for the assumed moderating effect of cognitive ability or social skills on interview success.

## Introduction

The interview is a prevalent tool in personnel selection (Wilk and Cappelli, 2003). Employers and applicants favor interviews because of their personal nature and because they give applicants the opportunity to display personal qualities (Anderson et al., 2010). However, when applicants use the opportunity to try to put their best foot forward, they are not always found to be completely honest. In fact, previous research has suggested that the prevalence of faking in interviews, on average, leads to more than two lies per interview (Weiss and Feldman, 2006), with over 90% of applicants admitting to having lied at least once during an interview (Levashina and Campion, 2007).

Furthermore, previous studies found that applicants differ in the amount of faking that they show during the whole selection process, including the interview (Donovan et al., 2003), which might lead to a change in the rank order of applicants. As a consequence, applicants who fake more frequently might improve their chances of receiving a job offer in comparison to applicants who provide relatively truthful answers. This makes faking in interviews a relevant issue for both practitioners and researchers because it could impair the interview’s criterion-related validity and eventually lead to suboptimal recruitment decisions, for example, when those who faked the most become the highest ranking applicants (cf., Donovan et al., 2014, for related research in the domain of personality testing).

Despite the high prevalence of faking behaviors and their potentially negative implications, two important issues remain largely unresolved. First, only a few previous studies have been devoted to antecedents of faking in interviews (e.g., Weiss and Feldman, 2006; Levashina and Campion, 2007; Hogue et al., 2012). According to faking theories (e.g., Snell et al., 1999; McFarland and Ryan, 2000, 2006; Levashina and Campion, 2007; Marcus, 2009; Roulin et al., 2016), there are several categories of antecedents that influence the occurrence of faking. These categories include applicants’ beliefs, attitudes, and personality, as well as their personal situation. However, empirical evidence that supports these antecedents in the interview domain is scarce. This lack of research is surprising because a deeper understanding of the factors that foster faking could serve as an initial indication of whether applicants are likely to fake or not.

Second, not all interviewees are successful with their faking behavior, which means that some might improve their interview scores or their chances of receiving a job offer through faking more than others, even though they are not lying or misrepresenting more in an absolute sense. We therefore have to distinguish between the mere occurrence of faking behavior and faking effectiveness. This is an important distinction because if faking does not improve interview scores, there might be less need to worry about the impairment of the psychometric properties of the interview. However, to the best of our knowledge, there has been no research so far that relates individual difference variables to faking effectiveness in interviews.

To address these gaps in our knowledge, we want to focus on two research questions in the present paper. First, what kind of individual difference variables foster the occurrence of faking behavior in interviews, and second, what kind of individual difference variables are relevant for faking effectiveness?

### Theoretical Background

Faking is defined as “an intentional distortion or a falsification of responses on measures in order to create a specific impression or provide the best answer” (Levashina and Campion, 2006, p. 300). Concerning faking in interviews, Levashina and Campion (2007) differentiated between four facets of faking behavior: slight image creation, extensive image creation, image protection, and ingratiation. Slight image creation can be seen as interviewees’ attempts to stretch the truth by overstating skills, abilities, or work experiences. When engaging in extensive image creation, interviewees invent (or borrow from others) work experiences, skills, or accomplishments. Image protection is used by interviewees to protect their image of being the ideal person for a certain job. For example, interviewees do not mention weaknesses and/or deficient skills, or they mask negative events and experiences from their past work history. Finally, deceptive ingratiation can be used by interviewees to become more likeable to the interviewer. This can be accomplished by pretending to conform to the interviewer’s and/or the organization’s values, beliefs, opinions, or attitudes, or by insincerely praising the interviewer or organization.

There are numerous models that deal with faking in general (Snell et al., 1999; McFarland and Ryan, 2000, 2006; Marcus, 2009; Roulin et al., 2016), and also specifically with faking in interviews (Levashina and Campion, 2006). Most of these models assume that features of the interviewee, such as their personality, attitudes, or abilities, as well as features of the situation, such as attractiveness of the position, influence the occurrence of faking.

Accordingly, Levashina and Campion’s (2006) model of faking likelihood in the employment interview conceptualizes faking as a function of capacity, willingness, and opportunity. All three of these factors have to be present to a certain degree for faking to occur. Capacity comprises skills and abilities, such as verbal skills, social skills, or cognitive ability. These skills and abilities help interviewees to identify the constructs being measured, to provide good answers to questions, and to act accordingly in order to boost their interview ratings. Willingness to fake comprises characteristics that influence the degree to which interviewees want to distort their response during an interview. It is influenced by interviewees’ characteristics, such as personality, motivation, and characteristics of the situation (e.g., a low probability of getting caught). Opportunity to fake can be given to interviewees, for example, by the type of questions someone is asked (e.g., strengths/weaknesses vs. job knowledge).

In the present study, we concentrate on capacity and willingness in order to gain a better understanding of the factors influencing faking that are rooted in the interviewee. We want to understand who is more likely to fake, and thereby also see whether we need to worry that faking might impair selection decisions, especially when unfavorable characteristics of the interviewee predict the occurrence of faking behavior.

The occurrence of faking behavior is predicted in the model by Levashina and Campion (2006). However, the model does not differentiate between faking occurrence and faking effectiveness. But as noted above, this is an important distinction that should be made. Variables such as personality characteristics and attitudes may determine whether someone does or does not fake, which is part of the abovementioned model by Levashina and Campion because these variables influence the motivation to fake. However, ability variables are probably relevant when it comes to faking effectiveness (i.e., to the issue of whether faking behavior actually leads to better interview evaluations or more job offers). Specifically, we follow Interpersonal Deception Theory (Buller and Burgoon, 1996, p. 224), which is based on the assumption that “skilled senders better convey a truthful demeanor by engaging in more strategic behavior and less non-strategic leakage than unskilled ones.” This means that fakers with higher abilities are better at distorting their answers such that they receive better ratings in interviews.

Both social and cognitive skills are necessary abilities that are needed for faking effectiveness. Interviews involve personal contact between interviewers and interviewees (Melchers et al., 2015). Accordingly, they involve spontaneous and flexible reactions to interviewers’ verbal and non-verbal behavior, and thus, interviewees who are more effective are probably those who are socially skilled. Furthermore, interviews place a high cognitive load on the interviewee (Van Iddekinge et al., 2005), which stresses the importance of particular skills or abilities (e.g., working memory, verbal ability) in order to fake successfully. Accordingly, interviewees need to monitor the interviewer closely, discern what the interviewer wants to hear, and adapt their answers accordingly. On top of that, the answers have to cover everything that the interviewer already knows about the interviewee, and has to withstand probing and follow-up questioning. In addition, interviewees only have a few seconds to prepare such an answer.

In line with this, in a study on faking in a biodata measure, Levashina et al. (2009) found that applicants with higher cognitive ability were less likely to fake the biodata measure, but when they did, they were more successful. This supports the assumption that cognitive skills have a positive influence on the quality of faking, and moderate the relationship between faking and interview success.

Levashina et al. (2009) supported their argument with further evidence from faking in non-cognitive measures. The results regarding the relation between cognitive ability and faking seem somewhat mixed at first (e.g., Ones et al., 1996; Lao, 2001; Pauls and Crost, 2005). However, Levashina et al. (2009) suggested that laboratory studies with instructions on how to fake well found positive correlations between cognitive ability and faking, whereas field studies found negative correlations. Levashina et al. (2009) argued that this is because applicants who are higher in cognitive ability do not need to fake because they have better chances of receiving a job offer in the first place. This is supported by the negative correlation between cognitive ability and faking in field studies (Ones et al., 1996; Boss et al., 2015). But when people fake (because, for example, they are instructed to do so), they are more skilled in doing so (e.g., they know which items or questions to fake, and in what way). This is supported by the positive correlation between cognitive ability and faking extent and faking quality in studies that used instructions to fake (e.g., Lao, 2001; Pauls and Crost, 2005).

Another stream of research that supports the moderating role of skills on successful self-presentation stemmed from research on political skills and self-monitoring. For example, Harris et al. (2007) found that political skills moderated the relationship between impression management on the job and supervisor ratings. Those individuals who used impression management tactics and were politically skilled received more favorable supervisor ratings than those who were not politically skilled. Similarly, Treadway et al. (2007) found that individuals who were highly politically skilled were less likely to have their ingratiation behavior on the job perceived as a manipulative influence attempt by their supervisors in comparison to individuals with low political skills. Finally, with regard to the role of self-monitoring, Turnley and Bolino (2001) found that individuals who were high in self-monitoring were more effective when using impression management strategies compared to individuals who were low in self-monitoring.

### Development of Hypotheses

We will first develop our hypotheses for faking occurrence and then for faking effectiveness. In the present study, we focus on the relationship between faking in interviews on the one hand, and attitudes and individual difference variables that seem especially relevant on the other hand. Specifically, these variables are attitude toward faking, honesty–humility, core self-evaluations (CSE), and Neuroticism, Conscientiousness, Extraversion, and Agreeableness of the Big Five. We chose these variables because of theoretical considerations and because of their relevance to the context of work (e.g., for team work or job performance in general).^1^ Thus, with regard to the Levashina and Campion (2006) model, we investigated personality variables and attitudes as antecedents that foster faking willingness, and therefore the occurrence of faking and cognitive ability and social skills, which are related to faking capacity.

#### Antecedents of Faking Occurrence

##### Attitude toward faking

Attitude toward faking refers to the degree to which an individual has a favorable or unfavorable view of faking. Positive attitude toward faking lead to intentions to show faking behavior. These, in turn, should lead to faking behavior according to McFarland and Ryan’s (2006) model of applicant faking behavior, which is based on the theory of planned behavior (Ajzen, 1991). According to McFarland and Ryan, attitude toward faking are a direct predictor of applicants’ intention to fake. In support of this suggestion, McFarland and Ryan found that attitude toward faking predicted intentions toward faking in personality tests, and also the actual degree of faking. It seems likely that the same kind of relationship should also hold true in the interview context. Accordingly, we posit the following hypothesis:

H1: A positive attitude toward faking is associated with more faking behavior.

##### Honesty–humility

Honesty–humility is a sixth personality dimension in the HEXACO model (Ashton et al., 2000; Ashton and Lee, 2009) that expands the five factor model (Costa and McCrae, 1992) by one factor. It consists of fairness, sincerity, modesty, and lack of greed, and it represents individual differences in the reluctance to exploit others (Lee and Ashton, 2004). We propose that people who are high in honesty–humility will fake less in interviews because honesty–humility is closely related to integrity (Lee et al., 2008), which, in turn, is associated with less applicant faking behavior (McFarland and Ryan, 2000). Honesty–humility also has a negative correlation with workplace delinquency, which is an indicator of unethical behavior, such as faking in interviews. Additionally, in their study with undergraduate students, Bourdage and Lee (2013) found that honesty–humility was the most consistent predictor of impression management in interviews. Thus, participants with high scores in honesty–humility were least likely to engage in impression management behavior. We therefore posit the following hypothesis:

H2: Honesty–humility is negatively associated with faking behavior.

##### Core self-evaluations

Core self-evaluations are an elementary assessment of oneself and one’s functioning in the world (Judge et al., 1997, 1998). They comprise the following four subfacets: self-esteem, (internal) locus of control, emotional stability, and generalized self-efficacy.

Core self-evaluations should be negatively associated with faking behavior because external locus of control, which is negatively associated with CSE, is related to more unethical decisions (Trevino and Youngblood, 1990), more academic dishonesty (Leming, 1980), and more tolerance toward cheating (Coleman and Mahaffey, 2000). Furthermore, individuals with fragile self-esteem are more likely to exaggerate in interviews (Kernis, 2003) because they possess low levels of self-concept clarity and therefore rely more on situational cues (Campbell et al., 1996). Those situational cues are given during an interview because interviews are strong situations (Mischel, 1973) and imply that one will present oneself in the best manner possible (e.g., through faking). We therefore posit the following hypothesis:

H3: Core self-evaluations are negatively related to faking behavior.

##### Big Five

In the following paragraphs, we develop hypotheses for dimensions of the Big Five and faking. We will concentrate on Neuroticism, Conscientiousness, Extraversion, and Agreeableness because these dimensions are theoretically and empirically linked to faking behavior. Especially, as mentioned above, the Big Five, and personality in general, are linked to faking willingness, which means that individuals with certain personality characteristics are more willing to fake compared to others. Below, we derive hypotheses for each of the Big Five regarding their relation with faking.

*Neuroticism* Neurotic individuals tend to worry about what others think about them (Costa and McCrae, 1992). Because of this, they are probably more likely to try to manage the impressions they make on other individuals. In doing so, they might also use deceptive forms of impression management.

Empirical evidence concerning the relationship between Neuroticism and faking has been presented by McFarland and Ryan (2000), as well as by Tonković (2012). In their laboratory study, McFarland and Ryan found that Neuroticism predicted faking in several non-cognitive measures. Similarly, Tonković found such a relationship between facets of Neuroticism and faking in personality tests. Furthermore, Neuroticism is also negatively related to intentions to fake in future job interviews (Lester et al., 2015) and to integrity (Ones, 1994, Unpublished), which, in turn—as already noted above—should have a negative effect on faking behavior (McFarland and Ryan, 2000). Therefore, we posit the following hypothesis:

H4: Neuroticism is positively associated with faking in interviews.

*Conscientiousness*. Conscientious individuals are rule abiding and responsible (McCrae and Costa, 1989). They should therefore fake less because faking would be against the rules of being honest and responsible, and it has also been argued that individuals who are high on Conscientiousness might feel uncomfortable when they do something that is in disagreement with these rules (McFarland and Ryan, 2000). In line with this, Tonković (2012), as well as McFarland and Ryan (2000), found negative correlations between faking and Conscientiousness for faking in non-cognitive tests, and Lester et al. (2015) found a similar negative correlation with intentions to fake future job interviews. We therefore posit the following hypothesis:

H5: Conscientiousness is negatively associated with faking in interviews.

*Extraversion*. Extraversion should be positively related to faking for two reasons. First of all, Extraversion is linked to ambition (Watson and Clark, 1997), which should encourage individuals to use a lot of means (including unethical ones) to achieve their goals. In line with this, Extraversion is related to overclaiming (Bing et al., 2011) and academic dishonesty (Anderman and Danner, 2008). Second, extraverts are more sociable. Related to this, Kashy and DePaulo (1996) pointed out that lying can serve important social interaction purposes, such as making others feel better or making oneself look better, and that these interaction purposes are of high relevance for extraverts. They also found that individuals with higher scores on Extraversion tend to lie more (Kashy and DePaulo, 1996). Accordingly, we predict:

H6: Extraversion is positively associated with faking in interviews.

*Agreeableness*. As agreeableness embraces the facets of modesty and straightforwardness/morality (Costa and McCrae, 1992), we believe that it should be negatively related to faking behavior because of conflict with other aspects, such as slight and extensive image creation. Furthermore, because of such agreeableness facets, agreeable individuals adhere more closely to social norms, such as the norm of being honest in order to maintain harmony. Finally, because of this, agreeableness is also negatively related to integrity (Ones et al., 1993). We therefore posit the following hypothesis:

H7: Agreeableness is negatively associated with faking in interviews.

#### Antecedents of Faking Effectiveness

##### Cognitive ability

As already noted earlier, successful faking in interviews places high cognitive demands on interviewees (Van Iddekinge et al., 2005), such as the need to adapt their answers to what interviewers want to hear or to monitor their non-verbal behavior. Therefore, there should be a direct effect of cognitive ability on faking success and—as a consequence of this—on interview success.

However, interviewees’ who are high in cognitive ability might feel a lower need to fake in the first place because they have better chances of receiving a job offer (e.g., because they have better grades and/or are more successful in their job). Yet, in line with several faking models (McFarland and Ryan, 2000; Levashina and Campion, 2006; Marcus, 2009), we assume a moderating effect of cognitive ability between faking and interview success. In comparison to interviewees with lower cognitive ability, those with higher cognitive ability are more successful when they show faking behavior in an interview. We therefore posit the following hypothesis:

H8: Cognitive ability moderates the relationship between faking behavior and interview success on the one hand, and interview success and faking effectiveness on the other hand, such that the relationship will be stronger when cognitive ability is high.

##### Social skills

Social skills comprise the ability of being sensitive to others’ communication, to monitor one’s communication to others, and to express messages effectively (Riggio, 1986). All these abilities are important in selection interviews as they help interviewees to adapt their behavior to more effectively influence the interviewer (Melchers et al., 2009). Indeed, meta-analyses found a positive relationship between social skills and interview performance (Salgado and Moscoso, 2002). On top of this, an experimental study by Riggio et al. (1987) found significant correlations between social skills and deception ability. Similarly, in a field study with applicants, Hogan et al. (2007) found correlations between faking in personality tests and social skills. This supports the argument that socially skilled individuals can more effectively control the impression they make on others. Therefore, we assume a direct, positive effect of social skills on interview success.

H9: Social skills are positively associated with interview success and faking effectiveness.

However, as mentioned in the earlier section on cognitive ability, interviewees who are high in social skills might feel less need to fake in the first place because they tend to be more successful in their jobs (Ferris et al., 2001). But when they do so, they are probably more successful because they are more skilled in influencing others successfully. We therefore posit a moderating effect in addition to the main effect:

H10: Social skills moderate the relationship between faking behavior and interview success on the one hand, and interview success and faking effectiveness on the other hand, such that the relationship will be stronger when social skills are high.

### Overview of the Studies

To investigate our hypotheses, we conducted two studies. Study 1 examined the effects of personality and attitudes on faking occurrence and the moderating influence of cognitive ability on interview success. And in Study 2 in which we further investigated the moderating effect of ability on the relationship between faking occurrence and faking effectiveness, we made use of an experimental setting to control for other variables that might have an effect on interview success, and therefore on faking effectiveness in actual interviews.

## Study 1

### Participants and Procedure

To determine the necessary sample size, we used G^∗^Power (Faul et al., 2009) to conduct a power analysis. We assumed an alpha level of 0.05, a desired power of 0.80, and a correlation of 0.20 or an interaction with an effect size of *f*^2^ = 0.08, which in both cases represents the midpoint between small to medium-sized effects, according to conventional standards (Cohen, 1992). The power analysis revealed that a sample size of 193 participants was necessary for the correlational test, and a sample size of 101 for the test of the moderator effect. Altogether, 222 participants took part in our study, so that the power of our analyses was sufficiently high to detect effects of small-to-medium size.

Of these participants, 157 were females and 65 were males. They were from all fields of work, but all of them took part in a distance learning program in psychology. Their age ranged from 18 to 67 years, with a mean of 31.89 years (*SD* = 10.27). All of them had interview experience with *M* = 7.15 interviews (*SD* = 7.89) on average. Participants were recruited through the online platform of the distance learning program. The participants voluntarily took part in the study to meet a course requirement.

Participants completed an online questionnaire that contained all study variables, which included questions related to faking in previous selection interviews, cognitive ability, the different non-cognitive individual difference variables, and attitudes (see below for more information).

Seventy-nine of the participants indicated that they only studied and did not currently work in a regular job. We therefore wanted to determine whether the relationships among the study variables were comparable for both samples. To do so, we conducted a χ^2^-test to evaluate the equality of the correlation matrices (Jennrich, 1970) of participants who studied full-time versus those who currently held a job and studied part-time. There was no significant difference between the matrices from the two subsamples, χ^2^(78) = 46.75, *p* = 0.99. We therefore conducted all later analyses with the whole sample.

### Measures

#### Cognitive Ability

Cognitive ability was assessed with the 10-Min Test by Musch et al. (2011, Unpublished). This test is a short test that assesses general intelligence within 10 min. It is comprised of 32 items that measure both fluid (e.g., numerical sequences) and crystallized cognitive ability [e.g., Which of the following fractions is the smallest? (1) 2/4; (2) 2/5; (3) 1/3; (4) 3/8]. Thus, similar to the Wonderlic Personnel Test (Wonderlic Inc, 2002), the items are taken from different ability domains with the aim of yielding an overall score for individuals’ general mental ability. In line with this, the 10-Min Test proved to be highly g saturated in studies by Ostapczuk et al. (2011) and Ostapczuk et al. (2014).

#### Honesty–Humility

Honesty–humility was measured with 16 items from the corresponding scale from the HEXACO questionnaire (Ashton and Lee, 2009). For this and all the following rating variables, items had to be answered on a 5-point rating scale from 1 (= *I do not agree at all*) to 5 (= *I fully agree*), unless indicated otherwise. The scale contains the four subfacets sincerity (“I wouldn’t use flattery to get a raise or promotion at work, even if I thought I would succeed”), fairness (“I would never accept a bribe, even if it were very large”), greed avoidance (“Having a lot of money is not especially important to me”), and modesty (“I am an ordinary person who is no better than others”). Cronbach’s alpha was 0.64 for sincerity, 0.72 for fairness, 0.79 for greed avoidance, and 0.69 for modesty.

#### Core Self-Evaluations

Core self-evaluations were measured with 12 items (e.g., “I am confident I get the success I deserve in life”) from the German version of Judge et al.’s (2003) CSE Scale (Stumpp et al., 2010). Cronbach’s alpha for this scale was 0.90.

#### Neuroticism

Neuroticism was measured with four items (e.g., “I see myself as someone who gets nervous easily”) from the short version of the Big Five Inventory (BFI-K, Rammstedt and John, 2005). Cronbach’s alpha was 0.84.

#### Conscientiousness

Conscientiousness was measured with four items (e.g., “I carry out tasks thoroughly”) from the short version of the Big Five Inventory (BFI-K, Rammstedt and John, 2005). Cronbach’s alpha was 0.75.

#### Extraversion

Extraversion was measured with four items (e.g., “I am an outgoing, sociable person”) from the short version of the Big Five Inventory (BFI-K, Rammstedt and John, 2005). Cronbach’s alpha was 0.89.

#### Agreeableness

Agreeableness was measured with four items (e.g., “I am a trusting person who believes in the good in man”) from the short version of the Big Five Inventory (BFI-K, Rammstedt and John, 2005). Cronbach’s alpha was 0.70.

#### Attitude toward Faking

Similar to McFarland and Ryan (2006), attitude toward faking was measured with a semantic differential. Participants had to indicate their attitude toward faking in interviews on 5-point semantic-differential type response scales: good–bad, appropriate–inappropriate, foolish–wise, useful–useless, and reasonable–unreasonable. Three items (good–bad, foolish–wise, useful–useless) were taken from McFarland and Ryan (2006), and the other two items (appropriate–inappropriate and reasonable–unreasonable) were developed for the present study. Cronbach’s alpha was 0.92.

#### Faking in Interviews

Faking in previous selection interviews was measured with 54 items from Levashina and Campion’s (2007) interview faking scale. This scale contains four dimensions with 11 facets. The four dimensions are slight image creation (e.g., “I exaggerated my responsibilities on my previous jobs”), extensive image creation (e.g., “I fabricated examples to show my fit with the organization”), image protection (e.g., “I did not reveal my true career intentions about working with the hiring organization”), and ingratiation (e.g., “I tried to agree with the interviewer outwardly even when I disagree inwardly”). When participants completed this scale, we asked them to think about their behavior in previous interviews. Cronbach’s alpha was 0.93 for slight image creation, 0.95 for extensive image creation, 0.91 for image protection, and 0.96 for ingratiation.

#### Interview Success

Interview success was operationalized as the ratio of the total number of successful interviews (i.e., job offers or making it to the next round) to the total number of previous interviews. Therefore, participants had to indicate how many interviews they had in the past and how many of these interviews were successful. We decided not to ask about the last interview because, as a single event, success in it could be affected by random influences, such as the interviewer, the type of job, the quality of other applicants, or other variables.

### Results

**Table 1** displays correlations, means, and standard deviations for all study variables. Given that the different subfacets of honesty–humility only showed moderate correlations with each other (mean *r* = 0.39), they were considered separately in the following analyses. In contrast to this, because of the relatively high correlations among the different facets of the faking questionnaire (mean *r* = 0.71), we calculated the average score across the different facets as an overall indicator of the degree to which individuals had faked in previous interviews.

**Table 1:** Descriptive statistics and intercorrelations of study variables for Study 1.

		*M*	*SD*	1	2	3	4	5	6	7	8	9	10	11	12	13
(1)	Self-reported faking behavior	1.79	0.66													
(2)	Interview success^a^	0.76	0.25	–0.03												
(3)	Attitude toward faking	2.94	1.10	0.58ˆ**	0.03											
(4)	Sincerity	3.41	0.84	–0.38ˆ**	–0.11	–0.40ˆ**										
(5)	Fairness	3.77	1.00	–0.45ˆ**	–0.01	–0.38ˆ**	0.44ˆ**									
(6)	Greed avoidance	3.75	0.86	–0.35ˆ**	–0.06	–0.33ˆ**	0.38ˆ**	0.40ˆ**								
(7)	Modesty	3.95	0.77	–0.41ˆ**	0.02	–0.29ˆ**	0.34ˆ**	0.32ˆ**	0.45ˆ**							
(8)	Core self-evaluations	3.66	0.65	–0.16ˆ*	0.17ˆ*	–0.11	0.00	0.08	0.09	0.10						
(9)	Neuroticism	2.75	0.93	0.13ˆ*	–0.09	–0.14ˆ*	–0.02	–0.10	–0.09	–0.07	–0.75ˆ**					
(10)	Conscientiousness	3.73	0.68	–0.04	0.12	–0.03	0.05	0.09	0.05	0.01	0.63ˆ**	–0.48ˆ**				
(11)	Extraversion	3.37	0.99	0.04	0.16ˆ*	0.00	–0.12	–0.03	0.00	0.03	0.51ˆ**	–0.42ˆ**	0.26ˆ**			
(12)	Agreeableness	3.29	0.76	–0.11	0.01	–0.20ˆ**	0.10	0.28ˆ**	0.20ˆ**	0.31ˆ**	0.25ˆ**	–0.34ˆ**	0.16ˆ*	0.26ˆ**		
(13)	Cognitive ability	18.14	4.43	–0.19ˆ**	–0.01	–0.10	0.06	0.09	0.14ˆ*	0.02	0.02	–0.05	–0.09	–0.07	–0.03	

*N = 222. ^a^Interview success was coded as the ratio of the number of successful interviews and the overall number of interviews. ^∗^*p* < 0.05, ^∗∗^*p* < 0.01 (two-tailed).*

Hypothesis 1 predicted that attitude toward faking is positively associated with faking in interviews. In line with this, attitude toward faking was negatively correlated with faking, *r* = 0.58, *p* < 0.01.

Hypothesis 2 predicted that honesty–humility is negatively associated with faking in interviews. In support of this, all correlations between subfacets of honesty–humility and faking in interviews were significant and in the expected direction. Specifically, we found negative correlations for sincerity, fairness, greed avoidance, and modesty with faking, *r* = -0.38, -0.45, -0.35, and -0.41, all *p*s < 0.01. Thus, our data provided strong support for the prediction that honesty–humility is negatively associated with faking in interviews.

Hypothesis 3 stated that CSE is negatively related to faking in interviews. In support of this, CSE correlated negatively with faking, *r* = -0.16, *p* < 0.05. Thus, individuals with higher scores on CSE reported less faking in interviews.

Hypothesis 4 to 7 predicted that dimensions of the Big Five namely Neuroticism, Conscientiousness, Extraversion, and Agreeableness are significantly related to faking in interviews. Only Neuroticism correlated positively with faking, *r* = 0.13, *p* < 0.05. For the other dimensions of the Big Five, these hypotheses were not supported given that none of the dimensions correlated significantly with the overall score for faking.

Hypothesis 8 predicted that cognitive ability moderates the relationship between faking behavior and interview success such that the relationship would be more positive when cognitive ability is high. We conducted a hierarchical multiple regression to test this hypothesis. To do so, we first mean centered the different predictors. Mean centering a variable means subtracting its overall mean from all its values and is a common procedure in moderated regression analysis (Cohen et al., 2013). In Step 1, we then entered faking behavior and cognitive ability. Neither faking behavior nor cognitive ability were significant predictors for interview success. In Step 2, we entered the interaction term of faking behavior and cognitive ability. In contrast to the prediction from Hypothesis 8, cognitive ability did not moderate the relation between faking behavior and interview success (see **Table 2**).

**Table 2:** Hierarchical regression analysis of cognitive ability as a moderator between faking and interview success.

	Interview success		
Model	β	*R*^2^	Δ*R*^2^
Step 1:		0.00	0.00
Self-reported faking behavior	–0.03	
Cognitive ability	–0.01		
Step 2:		0.00	0.00
Faking × Cognitive ability	–0.04	

*N = 222; Interview success was coded as the ratio of the number of successful interviews and number of interviews.*

Furthermore, given that Levashina and Campion (2006) conceptualized faking as consisting of different facets, we decided to repeat the analyses related to the previous hypotheses to see whether the different facets have different antecedents. Accordingly, we also determined correlations between the facets of the faking questionnaire and the other study variables. The correlational pattern remained largely the same, with only two exceptions: CSE correlated significantly with extensive image creation and image protection, *r*s = -0.17 and -0.19, respectively, both *p*s < 0.05, but not with the other facets, and Neuroticism correlated significantly with image protection *r* = 0.17, *p* < 0.05. For the moderator analysis, there was also no significant result when considering the facets separately.

### Discussion

As predicted, we found negative relationships between honesty–humility, CSE, and faking behavior. Similarly, Neuroticism and attitude toward faking were also significantly related to faking behavior. These findings are in line with faking theories (Levashina and Campion, 2006; McFarland and Ryan, 2006) and with previous empirical findings (McFarland and Ryan, 2006; Bourdage and Lee, 2013). However, it should be noted that the correlations for attitude toward faking and honesty–humility with faking behavior were considerably larger than the correlation for CSE. The high correlation between honesty–humility and attitudes are in line with past research (Lester et al., 2015; Law et al., 2016). This could be because they both are more proximal antecedents of faking than personality. Furthermore, only Neuroticism of the Big Five correlated significantly with faking behavior. An explanation for this could be that they are more distal antecedents than attitudes when it comes to faking. This is supported by the fact that correlations are also quite small in other studies on the relationship between the Big Five and self-reported faking behavior, (between 0.01 and 0.27; e.g., Lester et al., 2015; Roulin and Bourdage, 2017). In addition, we also did not find the predicted moderating influence of cognitive ability on the relationship between faking behavior and faking success.

## Study 2

Concerning the non-significant interaction between faking behavior and cognitive ability in Study 1, it might be possible that this result is due to the field setting, where we could not control for several other variables (e.g., type of interview, type of job for which participants had applied, etc.). Therefore, we had a closer look at the assumed moderator effect in a second data set in which we made use of a controlled laboratory setting and in which we also considered social skills. Initially, this data set was collected for a study that was concerned with the effects of faking in interviews on criterion-related validity, and the corresponding results are reported elsewhere (Buehl et al., 2016).

### Participants and Procedure

Participants were *N* = 108 undergraduate psychology students (91 females and 17 males) from a German university. Their age ranged from 18 to 39 years, with a mean of 22.69 (*SD* = 3.25). Most of them had previous interview experience (84.3%), with having participated in *M* = 3.56 (*SD* = 5.37) interviews on average. They were contacted via a mailing list of the psychology department. The student participants took part in the study to meet a course requirement, but voluntarily chose the present study among various others that were available. After they signed up for the study, participants were sent a link to an online questionnaire containing questions on demographic variables. After completing the questionnaire, participants were invited to take part in two structured interviews: one in an honest condition in which they were instructed to answer as honestly as possible, and the other one in a faking condition in which they were told to put their best foot forward and act as if they would be interviewed for a highly attractive graduate program. The order of the two interview conditions was counterbalanced across participants. To make sure that participants did not try to stick to the answers they gave in the first interview, there were at least 10 days between the first and the second interview. This is a common time period between the faking and honest condition that was also used in research on faking in personality inventories before (e.g., McFarland and Ryan, 2000; Dalen et al., 2001). We conducted panel interviews with two individuals on the panel. The first of them served as the interviewer and the second one as an additional rater. Each interviewer and each rater served on only one of the two conditions so that participants never met an interviewer or a rater twice. Cognitive ability was assessed before participants completed the first interview (see below for more information). Self-reported faking behavior was assessed after each interview.

### Faking Condition

For the faking condition, we tried to ensure that it resembled a real application situation as much as possible. Specifically, the interview was conducted in a conference room by a faculty member, participants were addressed formally by their last names, and both the interviewer and the participants were dressed in formal clothes that were suitable for an application process.

Before the interview, participants read the instruction that told them to put their best foot forward in order to appear as an ideal applicant for an attractive Master’s program in Psychology (in Germany, the majority of psychology students apply for a Master’s program because most employers request a Master’s degree for Psychology graduates). An interview as part of the admission process to a Master’s program is also common in Germany (Liste Master Psychologie, 2016). To increase the participants’ motivation to put their best foot forward, we informed them that 50 Euros (approximately 60 US dollars) would be given to the top 5% of the interviewees.

We chose an applicant-like faking condition because we deemed this as more realistic than a fake-good faking condition given that research on faking in personality tests revealed that actual applicants fake in a more reluctant way than participants in fake-good studies (see Viswesvaran and Ones, 1999; Birkeland et al., 2006), and also given that previous research on faking in interviews used such a condition (Van Iddekinge et al., 2005).

### Honest Condition

For the honest condition, we tried to ensure that participants answered as honestly as possible. We chose an informal setting (the interview was conducted in a departmental kitchen on comfortable chairs); both the interviewer as well as the interviewee were dressed as they would normally when attending university. The interviewer for this condition was an I/O Master’s student. Interviewees were addressed by their first name only. Before the interview, participants read the instruction, which told them that they were part of a study for an interview related to their study behavior, and therefore, they should answer as truthfully as possible. Furthermore, we told them that their answers would remain confidential and that we would use them for research purposes only.

### Measures

#### Structured Interview

The interview consisted of 20 questions (2 questions on career and program choice, 7 past behavior questions, 11 situational and future-oriented questions) and was designed to predict academic performance. The interview was developed from a large set of critical incidents from psychology students that were collected prior to this study. Critical incidents measured different behaviors necessary for academic success, such as teamwork, problem solving, conscientiousness, persistence, and planning and organizing. In a pilot study with *N* = 66 student participants, we found that the interview predicted university citizenship behavior, *r* = 0.49, *p* < 0.01, as well as peer-ratings of students’ academic performance, *r* = 0.29, *p* < 0.05.

All interviewers and raters underwent several hours of frame-of-reference training (Melchers et al., 2011; Roch et al., 2012). During the interview, the interviewer as well as the second rater took notes, and both provided independent ratings for each interview question on 5-point scales that contained behavioral rating anchors for 1 = *poor*, 3 = *moderate*, and 5 = *excellent* answers. When they differed two or more points in their rating, the interviewer and the second rater discussed their evaluations after the completion of the interview. Although the interviewer and the second rater did not have to agree with each other, most differences could be resolved after a short discussion so that the ratings for individual questions usually did not differ by more than one point after the discussion.

We calculated intraclass correlations (ICC 1.1) between the overall interview ratings (the mean across all 20 interview questions) in order to determine interrater reliability. The mean interrater reliability was 0.99 for the honest condition after discussion and 0.90 before discussion. For the faking condition, it was 0.96 after the discussion and 0.78 before this discussion. Pearson correlations between the two raters were comparable to these values, with 0.99 and 0.96 after discussion for the honest condition and the faking condition and 0.90 and 0.78 before discussion.

#### Self-Reported Faking Behavior

Self-reported faking behavior was measured as a manipulation check with Ingold et al.’s (2015) short version of Levashina and Campion’s (2007) interview faking scale. This short version contains 11 items that represent the 11 subfacets of the measure (e.g., “I have covered something up in order to be able to give better interview responses”). The items were answered on a 5-point rating scale ranging from 1 = *I do not agree at all* to 5 = *I fully agree*. Cronbach’s alpha for this scale was 0.88.

#### Faking Effectiveness

For operationalizing faking effectiveness, we used two different indicators. First, we used the regression-adjusted difference score (RADS) between the honest and the faking condition that was proposed by Burns and Christiansen (2011). This RADS avoids the psychometric problems that occur with using the raw difference score, namely that a difference score will always be positively related with the scores from the faking condition and negatively with scores from the honest condition. As was explained by Burns and Christiansen (2011, p. 361/362), regression-adjusted difference scores are “derived by regressing scores from the faking condition onto scores from the honest condition and computing the residual,” so that “the regression-adjusted difference score is interpreted as that part of the scores from the faking condition that cannot be explained by honest scores.”

As a second measure of faking effectiveness, we used the interview score from the faking condition in which participants were instructed to put their best foot forward.

#### Cognitive Ability

Cognitive ability was assessed with the Wonderlic (Wonderlic Inc, 2002). In this test, participants have to answer as many items as possible within 12 min. The 50-items test consists of questions on vocabulary, arithmetic reasoning, and spatial relations. The items are presented in order of increasing difficulty.

#### Social Skills

Social skills were measured with the ISK-K, the “Inventar sozialer Kompetenzen-Kurzversion” (the short version of the Inventory for Social Skills; Kanning, 2009). The ISK-K is a self-report measure that consists of the four subscales self-monitoring (“I am always successful in controlling my feelings”), offensiveness (“I love controversial discussions with other people”), reflexibility (“I always try to convey a positive picture about myself to other people”), and social orientation (“In most situations, I try to see the world through the eyes of my counterpart”). These subscales were derived by Kanning on the basis of extensive factor analytic work on a larger pool of items from the long version of the inventory. In the present sample, Cronbach’s alpha was 0.77 for self-monitoring, 0.55 for offensiveness, 0.63 for reflexibility, and 0.69 for social orientation.

### Results

To ensure that our experimental treatment worked as intended, we first analyzed data from the manipulation check. We found that participants in the faking condition had a significantly higher mean on the Interview Faking Behavior scale (*M* = 2.23, *SD* = 0.70) compared to the honest condition (*M* = 1.32, *SD* = 0.26), *t*(109) = 13.93 *p* < 0.01, *d* = 1.33 (*d* was calculated according to the formula by Cohen, 1988, for within-subjects data). Thus, participants in the faking condition indeed reported higher levels of faking behavior than in the honest condition.

**Table 3** displays correlations, means, and standard deviations for all study variables. Given that the different subscales from the social skills inventory only showed relatively small correlations with each other (mean *r* = 0.02), we refrained from combining them into an overall social skills score and instead considered them separately in the following analyses. As can be seen in **Table 3**, there were positive correlations between self-reported faking behavior and interview performance, *r* = 0.19, *p* < 0.05, and the regression-adjusted difference score, *r* = 0.31, *p* < 0.01, as well as a positive correlation between cognitive ability and the regression-adjusted difference score, *r* = 0.19, *p* < 0.05. The correlation between cognitive ability and interview performance fell short of significance, *r* = 0.17, *p* = 0.08. Finally, the two variables that were used as indicators of faking effectiveness (interview performance in the faking condition and the regression-adjusted difference score between the honest and the faking condition) showed a rather high correlation, *r* = 0.95, *p* < 0.01.

**Table 3:** Descriptive statistics and intercorrelations of study variables for Study 2.

		*M*	*SD*	1	2	3	4	5	6	7	8
(1)	Self-reported faking behavior	2.22	0.71								
(2)	Interview performance (faking condition)	3.62	0.25	0.19ˆ*							
(3)	Regression-adjusted difference score	0.00	0.24	0.31ˆ**	0.95ˆ**						
(4)	Cognitive ability	29.28	5.20	0.05	0.17	0.19ˆ*					
(5)	Self-monitoring	3.23	0.68	–0.11	0.09	0.06	0.12				
(6)	Offensiveness	3.36	0.51	–0.22ˆ*	–0.05	–0.09	0.04	0.20ˆ*			
(7)	Reflexibility	3.61	0.52	–0.10	0.05	0.05	–0.05	–0.38ˆ**	–0.03		
(8)	Social orientation	3.79	0.46	–0.27ˆ**	0.10	0.04	0.05	0.21ˆ*	0.07	0.04	

*N = 108. ^∗^*p* < 0.05, ^∗∗^*p* < 0.01 (two-tailed).*

Concerning our specific hypotheses, Hypothesis 8 stated that cognitive ability moderates the relationship between faking behavior and interview success with respect to faking effectiveness, such that the relationships will be stronger when cognitive ability is high. Similar to Study 1, we conducted a hierarchical multiple regression for each of the two dependent variables to test this hypothesis. Again, we mean-centered all predictors before the regression.

In Step 1, we entered faking behavior and cognitive ability. In line with the correlational results, faking behavior was a significant predictor for the RADS, β = 0.30, *p* < 0.05, but it fell short of significance as a predictor for the interview score in the faking condition, β = 0.18, *p* < 0.06. Furthermore, cognitive ability also fell short of significance as a predictor of faking effectiveness for both dependent variables: β = 0.16, *p* < 0.10, for the interview score in the faking condition and β = 0.18, *p* < 0.06, for the regression-adjusted difference score between the faking and the honest condition.

In Step 2, we entered the interaction term of faking behavior and cognitive ability. In contrast to our prediction, cognitive ability did not moderate the relationship between self-reported faking behavior and faking success for the two indicators of faking effectiveness, both βs < 0.06, both *p*s > 0.63 (see **Table 4**). Thus, the results did not provide support for Hypothesis 8.

**Table 4:** Results of hierarchical regression analyses of cognitive ability as a moderator between faking and faking effectiveness.

	Interview score			RADS		
Model	β	*R*^2^	Δ*R*^2^	β	*R*^2^	Δ*R*^2^
Step 1:		0.06	0.06		0.13^∗∗^	0.13^∗∗^
Self-reported faking behavior	0.18			0.30^∗^		
Cognitive ability	0.16			0.18		
Step 2:		0.06	0.00		0.13^∗∗^	0.00
Faking × Cognitive ability	0.05			0.00		

*N = 108; RADS = regression adjusted difference score. ^∗^*p* < 0.05, ^∗∗^*p* < 0.01 (two-tailed).*

Hypothesis 9 stated that social skills are positively related to faking effectiveness. This hypothesis was not supported given that none of the dimensions of social skills (self-monitoring, offensiveness, reflexibility, and social orientation) correlated significantly with the RADS or the interview score in the faking condition (i.e., with the two measures of faking effectiveness). Further inspection of **Table 3** also indicates that participants with higher scores on offensiveness and social orientation showed significantly less self-reported faking behavior, *r* = -0.22 and -0.27, both *p*s < 0.05, respectively, and that the same was true on a descriptive level for the other two social skills dimensions.

Finally, Hypothesis 10 stated that social skills moderate the relationship between faking behavior and interview success with respect to faking effectiveness such that the relationships will be stronger when social skills are high. We conducted hierarchical multiple regressions to test Hypothesis 10. Again, we mean-centered all predictors before calculating the interaction terms. In Step 1, we entered faking behavior and social skills (self-monitoring, offensiveness, reflexibility, and social orientation). Faking behavior was a significant predictor for the RADS, β = 0.36, *p* < 0.01, as well as for the interview score in the faking condition, β = 0.25, *p* < 0.05, but social skills was not a significant predictor for interview success. In Step 2, we entered the interaction terms of the social skills facets and faking behavior. Social skills did not moderate the relationship between faking behavior and faking effectiveness, and this was true for both dependent variables (see **Table 5**). Thus, these results did not provide support for Hypothesis 10.

**Table 5:** Results of hierarchical regression analyses of social skills as a moderator between faking and faking effectiveness.

	Interview score			RADS		
Model	β	*R*^2^	Δ*R*^2^	β	*R*^2^	Δ*R*^2^
Step 1:		0.08	0.08		0.13^∗^	0.13^∗^
Self-reported faking behavior	0.25ˆ*			0.35ˆ*		
Self-monitoring	0.14			0.14		
Offensiveness	–0.01			–0.04		
Reflexibility	0.06			0.13		
Social orientation	0.07			0.11		
Step 2:		0.12	0.04		0.16^∗^	0.03
Faking × Self-monitoring	–0.17			–0.15		
Faking × Offensiveness	0.12			0.09		
Faking × Reflexibility	–0.08			–0.05		
Faking × Social orientation	–0.07			–0.08		

*N = 108; RADS = regression adjusted difference score. ^∗^*p* < 0.05 (two-tailed).*

When we calculated all moderation analysis separately for the different facets of interview faking behavior, there were also no significant results.

## Discussion

In contrast to our predictions, we neither found a moderating effect of cognitive ability nor of social skills. There are several possible reasons for this. First of all, it might be that the moderating effect is rather small (also see the results from the biodata study by Levashina et al., 2009), so that we could not detect it with our sample size. Second, there might be restrictions of variance for cognitive ability as well as for faking behavior. Concerning the former, we tested only Psychology students, which causes a restriction in variance for cognitive ability because of the rather strict admission procedure to Psychology programs in Germany. In line with this, our sample had higher scores and a smaller standard deviation (*M* = 29.28, *SD* = 5.20) than the population norm (Wonderlic Inc, 2002) for the adult working population in general (*M* = 21.75, *SD* = 7.60). Furthermore, the likely restriction of variance for faking behavior stems from our experimental treatment, where we instructed all participants to put their best foot forward. Third, for the social skills measures in particular, Cronbach’s alpha was relatively low, with values between 0.77 and 0.55, which could also have contributed to the non-significant results. Another reason for the non-significant results could be the use of a self-report social skills measure. Future research could use ability tests instead of self-report scales for measuring social skills. Finally, the nature of the interview could have limited the influence of both faking and cognitive ability. Specifically, the interview in the present study was highly structured, and previous research found that structure reduces the impact of impression management in interviews (Barrick et al., 2009; Levashina et al., 2014) and also the correlation with cognitive ability (Huffcutt et al., 1996). Thus, future research that aims to test the moderating influence of cognitive ability and of social skills should also consider less structured interviews. Possibly, the chances of finding support for moderator effects are better in those interviews than in highly structured ones.

## General Discussion

We tried to answer the following two questions with our studies: First, what kind of individual difference variables foster the occurrence of faking behavior in interviews, and second, what kind of individual difference variables are relevant for faking effectiveness?

By doing so, we wanted to gain a better understanding of the influencing factors for faking that are rooted in the interviewee. We also tried to raise awareness of there being a difference between mere faking behavior, which is influenced by personality as well as attitudes, and faking effectiveness (e.g., raising interview ratings or getting a job offer by means of faking), which should be influenced by ability factors.

Concerning the first question, we found that individual difference variables, such as honesty–humility, Core-self evaluations, Neuroticism, and attitudes toward faking foster the occurrence of faking behavior. However, concerning the second question, we neither found support for the assumed moderator effect of cognitive ability or social skills on interview success nor did we find a direct effect of social skills. However, we found direct effects of cognitive ability. In Study 1, cognitive ability was negatively correlated with self-reported faking behavior, and in Study 2, cognitive ability was positively correlated with the regression-adjusted difference score between the honest and the faking condition, which was our proxy for faking effectiveness. In addition, the correlation between cognitive ability and the interview score in the faking condition fell short of significance, but the absolute value from our study (*r* = 0.17) nevertheless is rather close to the uncorrected meta-analytic value of 0.20 by Berry et al. (2007). We will later discuss these somewhat diverging results.

Our finding that personality variables and attitude are linked to faking behavior contribute to the question of who fakes in interviews, and are in line with previous research. Specifically, Roulin and Krings (2016) recently found that competitive worldviews, which can be seen as an attitude (Duckitt et al., 2002), also explained variance in faking beyond personality. Other research has also found significant correlations between faking behavior and personality. Specifically, individuals who were higher in Self-monitoring, Machiavellianism, and Extraversion tended to fake more in several studies (Weiss and Feldman, 2006; Levashina and Campion, 2007; Hogue et al., 2012), and similar to our results for honesty–humility, interviewees who were lower in integrity also reported faking much more (Levashina and Campion, 2007).

Concerning the null correlations between the social skill subscales and interview success in Study 2—and also concerning the negative correlations for two of the social skill subscales and self-reported faking behavior—we have to admit that we are not able to offer an explanation. For the self-monitoring scale, this result is even qualitatively opposite to earlier results that were mentioned in the previous paragraph. However, the limited internal consistency reliability of this measure might have added somewhat more measurement error than in other studies.

For the second research question (What kind of individual difference variables contribute to faking effectiveness?), we found no moderating effect in either study. However, in Study 2, there was some support for a direct effect of cognitive ability on faking effectiveness, which was operationalized as the regression-adjusted difference score and as the interview score in the faking condition. This result seemingly contradicts our results from Study 1, in which cognitive ability was not related to interview success. However, the positive relationship between cognitive ability and interview performance in Study 2 is in line with meta-analytic findings that found such a positive relationship (e.g., Berry et al., 2007). As Study 1 was a field study, a lot of different interviews went into our data with unknown circumstances that we could not control. Furthermore, we had different dependent variables in our two studies, namely the ratio of the number of successful interviews (i.e., job offers or making it to the next round) and the number of interviews as an indicator of interview success in Study 1 and interview performance and the regression-adjusted difference score in Study 2. Nevertheless, further research to investigate the relationship between cognitive ability and faking is necessary.

### Limitations and Lines for Future Research

Although we conducted two studies in order to compensate for the disadvantages that each study design entails, there are still some limitations. First, a limitation of Study 1 is that it used a cross-sectional design. Even though our study provides a first step concerning individual difference antecedents of faking, further research should also investigate such antecedents in a predictive design.

Second, a limitation of Study 2 is that it was conducted in a simulated setting. However, this was necessary to be able to experimentally control variables that we could not control for in Study 1 (e.g., interview questions, type of interview). Furthermore, we designed the faking condition to be as realistic as possible. We conducted the interviews in official rooms, instructed participants to be dressed like they would go to a selection interview, and the interviews were conducted by a faculty member. Furthermore, for the honest condition, it was important to conduct the interview in a protected environment to ensure that participants revealed the truth about themselves without fear of negative consequences.

Third, another possible limitation of Study 2 is that we used a student sample, though we adjusted the simulation of the application situation to the students’ situation. We designed our interview in order to predict academic success. As some German universities require interviews for the admission process for graduate programs, students took it as training for their real application. However, future research should conduct field studies in order to evaluate the external validity and generalizability of our results.

Fourth, another possible limitation of Study 2 can be the use of a highly structured interview (Campion et al., 1997), which might have limited the effects of faking. The reason for this is that Barrick et al. (2009) and Levashina et al. (2014) found that the more structured an interview is, the less it is susceptible to self-presentation tactics. So far, no research has investigated whether this is also true for the effects of faking, but given the clear pattern of results from Barrick et al. (2009) and from Levashina et al. (2014) it seems likely that interviewees can more easily influence their interview success in less structured interviews. Therefore, future research should investigate the effects of faking in such interviews.

Fifth, we used self-report measures to assess faking behavior in Studies 1 and 2. On the one hand, this is necessary because interviewers are hardly able to detect faking (Reinhard et al., 2013; Roulin et al., 2015) so that we needed to rely on what interviewees tell us about their behavior. On the other hand, future studies might also use more objective measures, such as bogus items for assessing faking behavior, or use another measure of faking in addition to self-report measures.

And finally, as noted above, we used an applicant instruction instead of a fake-good instruction in Study 2. It might be argued that this kind of instruction did not lead to faking behavior in all our interviewees, but instead to more honest impression management. Accordingly, Study 2 might underestimate the impact of the targeted moderators of the relationship between faking behavior and faking effectiveness. However, as was already argued above, we consider this a more realistic condition than a fake-good instruction. The reason for this is that research on faking in personality tests revealed that actual applicants fake in a more reluctant way than participants in fake-good studies (see Viswesvaran and Ones, 1999; Birkeland et al., 2006). To us, it seems likely that this is the case because individual differences to show more or less faking behavior can come into effect in application (or application-like) situations in comparison to situations in which all the participants are instructed to fake as much as possible. However, similar to the personality domain, it might be possible that future research reveals evidence that the mean difference between a faking condition and an honest condition is larger when a fake-good instruction is used than when an application instruction is used.

### Implications

We examined the extent to which attitudes and individual difference variables are related to faking in interviews. Especially the results from Study 1 that individuals with low scores on honesty–humility reported more faking behavior might seem worrisome at first. Together with other findings that individuals with negative personality attributes show more faking behavior (Weiss and Feldman, 2006; Levashina and Campion, 2007; Hogue et al., 2012), this suggests that applicants with attributes that are also predictive of various other forms of unethical behavior are more prone to try to improve their chances in a selection interview.

However, before organizations become concerned about applicant faking in selection interviews, it is important to bear in mind the difference between faking occurrence and faking effectiveness. Not all interviewees are successful with their faking behavior, which means that some might improve their interview scores or their chances for a job offer through faking more than others. This is an important distinction because if faking does not improve interview scores, there might be less need to worry about the impairment of psychometric properties of the interview.

Accordingly, the non-significant relationships between self-reported faking behavior and interview success in our field study might be seen as comforting. Furthermore, these findings are mostly in line with previous research from two other field studies that examined the relationship between self-reported faking behavior and interview success. In line with our results, Roulin et al. (2014) found no significant relationship between self-reported faking behavior and interview performance, and Levashina and Campion (2007) found no significant relationship between slight image creation, image protection, and ingratiation (three of the dimensions of their interview faking scale), but only one with extensive image creation (the fourth dimension of this scale). In contrast to this, however, we found a positive correlation between faking behavior and interview performance in Study 2. However, in this study, we instructed participants to put their best foot forth. Furthermore, the significant correlation of interview performance with self-reported faking behavior in this study was accompanied by a larger relationship with cognitive ability, which is generally considered as beneficial for criterion-related validity (Schmidt and Hunter, 1998).

## Conclusion

Based on the available evidence, it seems premature to conclude that there is no need for organizations or interviewers to worry with regard to the effects of faking in interviews. However, if one considers our results and the results from previous research together, faking in interviews does not seem to pose a great danger to selection decisions because it is not significantly related to interview success in participants’ prior selection interviews. Nevertheless, future research is still needed in order to examine this relationship closer and to accumulate more evidence that also considers criterion-related validity to see whether faking impairs the quality of selection decisions based on the interview.

## Ethics Statement

In Germany, approval by an ethics committee is only necessary for psychological research if medical or physiological information about the participants are collected or when participants are treated in a way that may harm their health. As we did not use such kind of data or apply such kind of treatment in our study, we did not need to get approval from the ethics committee. However, we closely followed the guidelines for the treatment of human participants of the German Psychological Association.

In each study, we informed participants about purpose, duration, and procedures of the study and about the confidentiality of all the data that were collected. Furthermore, they were informed that they could always withdraw from the study without any disadvantages, about incentives of participation, and whom they could contact about questions. After they had received this information they agreed to participate in our study and also that data from them was collected, stored, and analyzed for our research.

## Author Contributions

A-KB and KM made substantial contributions to the conception and design of the studies. A-KB was responsible for collecting and analyzing the data, and drafting the paper and A-KB and KM both revised it critically for important intellectual content.

## Conflict of Interest Statement

The authors declare that the research was conducted in the absence of any commercial or financial relationships that could be construed as a potential conflict of interest.

## Footnotes

1 We chose to include Neuroticism separately even though it is also part of CSE. The reason for this was that we wanted to examine all relevant dimensions of the Big Five, but at the same to also include CSE because of its relevance in the occupational context.

## References

[B1] Ajzen I. (1991). The theory of planned behavior. *Organ. Behav. Hum. Decis. Process.* 50 179–211. 10.1016/0749-5978(91)90020-T

[B2] Anderman E. Danner F. (2008). Achievement goals and academic cheating. *Rev. Int. Psychol. Soc.* 21 155–180. 10.1177/0956797613487221

[B3] Anderson N. Salgado J. F. Hülsheger U. R. (2010). Applicant reactions in selection: comprehensive meta-analysis into reaction generalization versus situational specificity. *Int. J. Sel. Assess.* 18 291–304. 10.1111/j.1468-2389.2010.00512.x

[B4] Ashton M. C. Lee K. (2009). The HEXACO-60: a short measure of the major dimensions of personality. *J. Pers. Assess.* 91 340–345. 10.1080/00223890902935878 20017063

[B5] Ashton M. C. Lee K. Son C. (2000). Honesty as the sixth factor of personality: correlations with machiavellianism, primary psychopathy, and social adroitness. *Eur. J. Pers.* 14 359–368. 10.1002/1099-0984(200007/08)14:4<359::AID-PER382>3.0.CO;2-Y

[B6] Barrick M. R. Shaffer J. A. DeGrassi S. W. (2009). What you see may not be what you get: relationships among self-presentation tactics and ratings of interview and job performance. *J. Appl. Psychol.* 94 1394–1411. 10.1037/a0016532 19916651

[B7] Berry C. M. Sackett P. R. Landers R. N. (2007). Revisting interview-cognitive ability relationships: attending to specific range restriction mechanisms in meta-analysis. *Pers. Psychol.* 60 837–874. 10.1111/j.1744-6570.2007.00093.x

[B8] Bing M. N. Kluemper D. Davison H. K. Taylor S. Novicevic M. (2011). Overclaiming as a measure of faking. *Organ. Behav. Hum. Decis. Process.* 116 148–162. 10.1016/j.obhdp.2011.05.006

[B9] Birkeland S. A. Manson T. M. Kisamore J. L. Brannick M. T. Smith M. A. (2006). A meta-analytic investigation of job applicant faking on personality measures. *Int. J. Sel. Assess.* 14 317–335. 10.1111/j.1468-2389.2006.00354.x

[B10] Boss P. König C. J. Melchers K. G. (2015). Faking good and faking bad among military conscripts. *Hum. Perform.* 28 26–39. 10.1080/08959285.2014.974758

[B11] Bourdage J. S. Lee K. (2013). An investigation of personality, impression management, and interview performance. *Paper Presented at the 28th Annual Conference of the Society for Industrial and Organisational Psychology* Houston, TX.

[B12] Buehl A.-K. Melchers K. G. Macan T. Körner B. (2016). How does faking affect the psychometric properties of selection interviews? *Paper Presented at the 31st Annual Conference of the Society of Industrial and Organizational Psychology* Anaheim, CA.

[B13] Buller D. B. Burgoon J. K. (1996). Interpersonal deception theory. *Commun. Theory* 6 203–242. 10.1111/j.1468-2885.1996.tb00127.x

[B14] Burns G. N. Christiansen N. D. (2011). Methods of measuring faking behavior. *Hum. Perform.* 24 358–372. 10.1080/08959285.2011.597473

[B15] Campbell J. D. Trapnell P. D. Heine S. J. Katz I. M. Lavallee L. F. Lehman D. R. (1996). Self-concept clarity: measurement, personality correlates, and cultural boundaries. *J. Pers. Soc. Psychol.* 70 141–156. 10.1037/0022-3514.70.1.141

[B16] Campion M. A. Palmer D. K. Campion J. E. (1997). A review of structure in the selection interview. *Pers. Psychol.* 50 655–702. 10.1111/j.1744-6570.1997.tb00709.x

[B17] Cohen J. (1988). *Statistical Power Analysis for the Behavioral Sciences* 2nd Edn. Hillsdale, NJ: Erlbaum.

[B18] Cohen J. (1992). A power primer. *Psychol. Bull.* 112 155–159. 10.1037/0033-2909.112.1.155 19565683

[B19] Cohen J. Cohen P. West S. G. Aiken L. S. (2013). *Applied Multiple Regression/Correlation Analysis for the Behavioral Sciences.* New York, NY: Routledge.

[B20] Coleman N. Mahaffey T. (2000). Business student ethics: selected predictors of attitudes toward cheating. *Teach. Bus. Ethics* 4 121–136. 10.1023/A:1009855128668

[B21] Costa P. T. McCrae R. R. (1992). Four ways five factors are basic. *Pers. Individ. Dif.* 13 653–665. 10.1016/0191-8869(92)90236-I

[B22] Dalen L. H. Stanton N. A. Roberts A. D. (2001). Faking personality questionnaires in personnel selection. *J. Manage. Dev.* 20 729–742. 10.1108/02621710110401428

[B23] Donovan J. J. Dwight S. A. Hurtz G. M. (2003). An assessment of the prevalence, severity, and verifiability of entry-level applicant faking using the randomized response technique. *Hum. Perform.* 16 81–106. 10.1207/S15327043HUP1601_4

[B24] Donovan J. J. Dwight S. A. Schneider D. (2014). The impact of applicant faking on selection measures, hiring decisions, and employee performance. *J. Bus. Psychol.* 29 479–493. 10.1007/s10869-013-9318-5

[B25] Duckitt J. Wagner C. du Plessis I. Birum I. (2002). The psychological bases of ideology and prejudice: testing a dual process model. *J. Pers. Soc. Psychol.* 83 75–93. 10.1037/0022-3514.83.1.75 12088134

[B26] Faul F. Erdfelder E. Buchner A. Lang A.-G. (2009). Statistical power analyses using G^∗^Power 3.1: tests for correlation and regression analyses. *Behav. Res. Methods* 41 1149–1160. 10.3758/BRM.41.4.1149 19897823

[B27] Ferris G. R. Witt L. A. Hochwarter W. A. (2001). Interaction of social skill and general mental ability on job performance and salary. *J. Appl. Psychol.* 86 1075–1082. 10.1037/0021-9010.86.6.1075 11768051

[B28] Harris K. J. Kacmar K. M. Zivnuska S. Shaw J. D. (2007). The impact of political skill on impression management effectiveness. *J. Appl. Psychol.* 92 278–285. 10.1037/0021-9010.92.1.278 17227169

[B29] Hogan J. Barrett P. Hogan R. (2007). Personality measurement, faking, and employment selection. *J. Appl. Psychol.* 92 1270–1285. 10.1037/0021-9010.92.5.1270 17845085

[B30] Hogue M. Levashina J. Hang H. (2012). Will I fake it? The interplay of gender, Machiavellianism, and self-monitoring on strategies for honesty in job interviews. *J. Bus. Ethics* 117 1–13. 10.1007/s10551-012-1525-x

[B31] Huffcutt A. I. Roth P. L. McDaniel M. A. (1996). A meta-analytic investigation of cognitive ability in employment interview evaluations: moderating characteristics and implications for incremental validity. *J. Appl. Psychol.* 81 459–473. 10.1037/0021-9010.81.5.459

[B32] Ingold P. V. Kleinmann M. König C. J. Melchers K. G. (2015). Shall we continue or stop disapproving of self-presentation? Evidence on impression management and faking in a selection context and their relation to job performance. *Eur. J. Work Organ. Psychol.* 24 420–432. 10.1080/1359432X.2014.915215

[B33] Jennrich R. I. (1970). An asymptotic χ^2^ test for the equality of two correlation matrices. *J. Am. Stat. Assoc.* 65 904–912. 10.2307/2284596

[B34] Judge T. A. Erez A. Bono J. E. (1998). The power of being positive: the relation between positive self-concept and job performance. *Hum. Perform.* 11 167–187. 10.1080/08959285.1998.9668030

[B35] Judge T. A. Erez A. Bono J. E. Thoresen C. J. (2003). The core self-evaluations scale: development of a measure. *Pers. Psychol.* 56 303–331. 10.1111/j.1744-6570.2003.tb00152.x

[B36] Judge T. A. Locke E. A. Durham C. C. (1997). The dispositional causes of job satisfaction: a core evaluations approach. *Res. Organ. Behav.* 19 151–188.

[B37] Kanning U. P. (2009). *ISK- Inventar Sozialer Kompetenzen. [Inventory for Social Skills].* Göttingen: Hogrefe.

[B38] Kashy D. A. DePaulo B. M. (1996). Who lies? *J. Pers. Soc. Psychol.* 70 1037–1051. 10.1037/0022-3514.70.5.1037 8656334

[B39] Kernis M. H. (2003). Toward a conceptualization of optimal self-esteem. *Psychol. Inq.* 14 1–26. 10.1207/S15327965PLI1401_01

[B40] Lao R. Y.-R. (2001). Faking in personality measures: effects on the prediction of job performance. *Diss. Abstr. Int. B Sci. Eng.* 62 586–594.

[B41] Law S. J. Bourdage J. O’Neill T. A. (2016). To fake or not to fake: antecedents to interview faking, warning instructions, and its impact on applicant reactions. *Front. Psychol.* 7:1771 10.3389/fpsyg.2016.01771 PMC5108801 27895609

[B42] Lee K. Ashton M. C. (2004). Psychometric properties of the HEXACO personality inventory. *Multivar. Behav. Res.* 39 329–358. 10.1207/s15327906mbr3902_8 26804579

[B43] Lee K. Ashton M. C. Morrison D. L. Cordery J. Dunlop P. D. (2008). Predicting integrity with the HEXACO personality model: use of self-and observer reports. *J. Occup. Organ. Psychol.* 81 147–167. 10.1348/096317907X195175

[B44] Leming J. S. (1980). Cheating behavior, subject variables, and components of the internal-external scale under high and low risk conditions. *J. Educ. Res.* 74 83–87. 10.1080/00220671.1980.10885288

[B45] Lester C. Anglim J. Fullarton C. (2015). Individual differences in intention to fake job interviews: personality, self-monitoring, and the theory of planned behaviour. *Australas. J. Organ. Psychol.* 8 1–11. 10.1017/orp.2015.7

[B46] Levashina J. Campion M. A. (2006). A model of faking likelihood in the employment interview. *Int. J. Sel. Assess.* 14 299–316. 10.1111/j.1468-2389.2006.00353.x

[B47] Levashina J. Campion M. A. (2007). Measuring faking in the employment interview: development and validation of an interview faking behavior scale. *J. Appl. Psychol.* 92 1638–1656. 10.1037/0021-9010.92.6.1638 18020802

[B48] Levashina J. Hartwell C. J. Morgeson F. P. Campion M. A. (2014). The structured employment interview: narrative and quantitative review of the research literature. *Pers. Psychol.* 67 241–293. 10.1111/peps.12052

[B49] Levashina J. Morgeson F. P. Campion M. A. (2009). They don’t do it often, but they do it well: exploring the relationship between applicant mental abilities and faking. *Int. J. Sel. Assess.* 17 271–281. 10.1111/j.1468-2389.2009.00469.x

[B50] Liste Master Psychologie (2016). Available at: http://www.psystudents.org/wp-content/uploads/2014/03/Deutschland_Master-Psychologie-in-Deutschland-2016-mit-Fristen_Version_1.0.pdf [accessed 20 March, 2016].

[B51] Marcus B. (2009). ‘Faking’ from the applicant’s perspective: a theory of self-presentation in personnel selection settings. *Int. J. Sel. Assess.* 17 417–430. 10.1111/j.1468-2389.2009.00483.x

[B52] McCrae R. R. Costa P. T. (1989). The structure of interpersonal traits: Wiggins’s circumplex and the five-factor model. *J. Pers. Soc. Psychol.* 56 586–595. 10.1037/0022-3514.56.4.586 2709308

[B53] McFarland L. A. Ryan A. M. (2000). Variance in faking across noncognitive measures. *J. Appl. Psychol.* 85 812–821. 10.1037/0021-9010.85.5.812 11055152

[B54] McFarland L. A. Ryan A. M. (2006). Toward an integrated model of applicant faking behavior. *J. Appl. Soc. Psychol.* 36 979–1016. 10.1111/j.0021-9029.2006.00052.x

[B55] Melchers K. G. Ingold P. V. Wilhelmy A. Kleinmann M. (2015). “Beyond validity: shedding light on the social situation in employment interviews,” in *Employee Recruitment, Selection, and Assessment: Contemporary Issues for Theory and Practice* eds Nikolaou I. Oostrom J. K. (Hove: Psychology Press) 154–171.

[B56] Melchers K. G. Klehe U.-C. Richter G. M. Kleinmann M. König C. J. Lievens F. (2009). “I know what you want to know”: the impact of interviewees’ ability to identify criteria on interview performance and construct-related validity. *Hum. Perform.* 22 355–374. 10.1080/08959280903120295

[B57] Melchers K. G. Lienhardt N. von Aarburg M. Kleinmann M. (2011). Is more structure really better? A comparison of frame-of-reference training and descriptively anchored rating scales to improve interviewers’ rating quality. *Pers. Psychol.* 64 53–97. 10.1111/j.1744-6570.2010.01202.x

[B58] Mischel W. (1973). Toward a cognitive social learning reconceptualization of personality. *Psychol. Rev.* 80 252–283. 10.1037/h0035002 4721473

[B59] Ones D. S. Viswesvaran C. Reiss A. D. (1996). Role of social desirability in personality testing for personnel selection: the red herring. *J. Appl. Psychol.* 81 660–679. 10.1037/0021-9010.81.6.660

[B60] Ones D. S. Viswesvaran C. Schmidt F. L. (1993). Comprehensive meta-analysis of integrity test validities: findings and implications for personnel selection and theories of job performance. *J. Appl. Psychol.* 78 679–703. 10.1037/0021-9010.78.4.679

[B61] Ostapczuk M. Musch J. Lieberei W. (2011). Der “Analytische Test”: Validierung eines neuen eignungsdiagnostischen Instruments zur Erfassung von schlussfolgerndem Denken [The Analytische Test: validation of a new personnel selection tool assessing reasoning]. *Z. Arbeits Organisationspsychol.* 55 1–16. 10.1026/0932-4089/a000031

[B62] Ostapczuk M. Wagner M. Musch J. (2014). Revisiting the Rybakov figures: a classical and probabilistic analysis of the psychometric properties of a test of spatial visualization. *Swiss J. Psychol.* 73 57–67. 10.1024/1421-0185/a000125

[B63] Pauls C. A. Crost N. W. (2005). Cognitive ability and self-reported efficacy of self-presentation predict faking on personality measures. *J. Individ. Dif.* 26 194–206. 10.1027/1614-0001.26.4.194

[B64] Rammstedt B. John O. P. (2005). Kurzversion des Big Five Inventory (BFI-K) [Short version of the Big Five Inventory (BFI-K)]. *Diagnostica* 51 195–206. 10.1026/0012-1924.51.4.195

[B65] Reinhard M.-A. Scharmach M. Müller P. (2013). It’s not what you are, it’s what you know: experience, beliefs, and the detection of deception in employment interviews. *J. Appl. Soc. Psychol.* 43 467–479. 10.1111/j.1559-1816.2013.01011.x

[B66] Riggio R. E. (1986). Assessment of basic social skills. *J. Pers. Soc. Psychol.* 51 649–660. 10.1037/0022-3514.51.3.649

[B67] Riggio R. E. Tucker J. Throckmorton B. (1987). Social skills and deception ability. *Pers. Soc. Psychol. Bull.* 13 568–577. 10.1177/0146167287134013

[B68] Roch S. G. Woehr D. J. Mishra V. Kieszczynska U. (2012). Rater training revisited: an updated meta-analytic review of frame-of-reference training. *J. Occup. Organ. Psychol.* 85 370–395. 10.1111/j.2044-8325.2011.02045.x

[B69] Roulin N. Bangerter A. Levashina J. (2014). Interviewers’ perceptions of impression management in employment interviews. *J. Manag. Psychol.* 29 141–163. 10.1108/JMP-10-2012-0295

[B70] Roulin N. Bangerter A. Levashina J. (2015). Honest and deceptive impression management in the employment interview: can it be detected and how does it impact evaluations? *Pers. Psychol.* 68 395–444. 10.1111/peps.12079

[B71] Roulin N. Bourdage J. S. (2017). Once an impression manager, always an impression manager? Antecedents of honest and deceptive impression management use and variability across multiple job interviews. *Front. Psychol.* 8:29 10.3389/fpsyg.2017.00029 PMC5258756 28174546

[B72] Roulin N. Krings F. (2016). When winning is everything: the relationship between competitive worldviews and job applicant faking. *Appl. Psychol.* 65 643–670. 10.1111/apps.12072

[B73] Roulin N. Krings F. Binggeli S. (2016). A dynamic model of applicant faking. *Organ. Psychol. Rev.* 6 145–170. 10.1177/2041386615580875

[B74] Salgado J. F. Moscoso S. (2002). Comprehensive meta-analysis of the construct validity of the employment interview. *Eur. J. Work Organ. Psychol.* 11 299–324. 10.1080/13594320244000184

[B75] Schmidt F. L. Hunter J. E. (1998). The validity and utility of selection methods in personnel psychology: practical and theoretical implications of 85 years of research findings. *Psychol. Bull.* 124 262–274. 10.1037/0033-2909.124.2.262

[B76] Snell A. F. Sydell E. J. Lueke S. B. (1999). Towards a theory of applicant faking: integrating studies of deception. *Hum. Resour. Manage. Rev.* 9 219–242. 10.1016/S1053-4822(99)00019-4

[B77] Stumpp T. Muck P. M. Hülsheger U. R. Judge T. A. Maier G. W. (2010). Core self-evaluations in Germany: validation of a German measure and its relationships with career success. *Appl. Psychol. Int. Rev.* 59 674–700. 10.1111/j.1464-0597.2010.00422.x

[B78] Tonković M. (2012). Are there personality traits that predispose applicants to fake noncognitive measures in personnel selection? *Rev. Psychol.* 19 29–36.

[B79] Treadway D. C. Ferris G. R. Duke A. B. Adams G. L. Thatcher J. B. (2007). The moderating role of subordinate political skill on supervisors’ impressions of subordinate ingratiation and ratings of subordinate interpersonal facilitation. *J. Appl. Psychol.* 92 848–855. 10.1037/0021-9010.92.3.848 17484564

[B80] Trevino L. K. Youngblood S. A. (1990). Bad apples in bad barrels: a causal analysis of ethical decision-making behavior. *J. Appl. Psychol.* 75 378–385. 10.1037/0021-9010.75.4.378

[B81] Turnley W. H. Bolino M. C. (2001). Achieving desired images while avoiding undesired images: exploring the role of self-monitoring in impression management. *J. Appl. Psychol.* 86 351–360. 10.1037/0021-9010.86.2.351 11393446

[B82] Van Iddekinge C. H. Raymark P. H. Roth P. L. (2005). Assessing personality with a structured employment interview: construct-related validity and susceptibility to response inflation. *J. Appl. Psychol.* 90 536–552. 10.1037/0021-9010.90.3.536 15910148

[B83] Viswesvaran C. Ones D. S. (1999). Meta-analyses of fakability estimates: implications for personality measurement. *Educ. Psychol. Meas.* 59 197–210. 10.1177/00131649921969802

[B84] Watson D. Clark L. A. (1997). “Extraversion and its positive emotional core,” in *Handbook of Personality Psychology* eds Hogan R. Johnson J. A. Briggs S. R. (San Diego, CA: Academic Press) 767–793.

[B85] Weiss B. Feldman R. S. (2006). Looking good and lying to do it: deception as an impression management strategy in job interviews. *J. Appl. Soc. Psychol.* 36 1070–1086. 10.1111/j.0021-9029.2006.00055.x

[B86] Wilk S. L. Cappelli P. (2003). Understanding the determinants of employer use of selection methods. *Pers. Psychol.* 56 103–124. 10.1111/j.1744-6570.2003.tb00145.x

[B87] Wonderlic Inc (2002). *Wonderlic Personnel Test and Scholastic Level Exam: User’s Manual.* Libertyville, IL: Author.

